# Does HIV index testing bring patients into treatment at earlier stages of HIV disease? Results from a retrospective study in Ukraine

**DOI:** 10.1186/s12879-024-09190-7

**Published:** 2024-03-18

**Authors:** Andrew M. Secor, Alyona Ihnatiuk, Anna Shapoval, Misti McDowell, Larisa Hetman, Anjuli D. Wagner, Jillian Pintye, Kristin Beima-Sofie, Matthew R. Golden, Nancy Puttkammer

**Affiliations:** 1 Department of Global Health, University of Washington, Hans Rosling Center, 3980 15th Ave NE, 98105 Seattle, WA USA; 2 International Training and Education Center for Health (I-TECH), Kyiv, Ukraine; 3 International Training and Education Center for Health (I-TECH), Seattle, WA USA; 4 Public Health Center (PHC) of the Ministry of Health (MoH) of Ukraine, Kyiv, Ukraine; 5 Department of Medicine, University of Washington, Seattle, WA USA; 6 HIV/STD Program, Public Health-Seattle & King County, Seattle, WA USA

**Keywords:** HIV care continuum, HIV testing, Differentiated care, Index testing services, Linkage to care, Ukraine

## Abstract

**Background:**

Over one-third of people living with HIV (PLH) in Ukraine are not on treatment. Index testing services, which link potentially exposed partners (named partners) of known PLH (index patients) with testing and treatment services, are being scaled in Ukraine and could potentially close this gap.

**Methods:**

This retrospective study included patient data from 14,554 adult PLH who initiated antiretroviral treatment (ART) between October 2018 and May 2021 at one of 35 facilities participating in an intervention to strengthen index testing services. Mixed effects modified Poisson models were used to assess differences between named partners and other ART initiators, and an interrupted time series (ITS) analysis was used to assess changes in ART initiation over time.

**Results:**

Compared to other ART initiators, named partners were significantly less likely to have a confirmed TB diagnosis (aRR = 0.56, 95% CI = 0.40, 0.77, *p* < 0.001), a CD4 count less than 200 cells/mm^3^ (aRR = 0.84, 95% CI = 0.73, 0.97, *p* = 0.017), or be categorized as WHO HIV stage 4 (aRR = 0.68, 9% CI = 0.55, 0.83, *p* < 0.001) at the time of ART initiation, and were significantly more likely to initiate ART within seven days of testing for HIV (aRR = 1.36, 95% CI = 1.22, 1.50, *p* < 0.001). Our ITS analysis showed a modest 2.34% (95% CI = 0.26%, 4.38%; *p* = 0.028) month-on-month reduction in mean ART initiations comparing the post-intervention period to the pre-intervention period, although these results were likely confounded by the COVID epidemic.

**Conclusion:**

Our findings suggest that index testing services may be beneficial in bringing PLH into treatment at an earlier stage of HIV disease and decreasing delays between HIV testing and ART initiation, potentially improving patient outcomes and retention in the HIV care cascade.

**Supplementary Information:**

The online version contains supplementary material available at 10.1186/s12879-024-09190-7.

## Background

HIV remains a major public health concern in Ukraine, with an estimated 245,000 cases and an incidence of 0.21 per 1,000 person-years at risk in 2021 [1, 2]. Prevention efforts have been hindered by low rates of testing and treatment; it is estimated that only 75% of people living with HIV (PLH) in Ukraine know their status, 83% aware of their status are receiving treatment, and 94% on treatment are virally suppressed [2]. Testing and treatment coverage are even lower among hard-to-reach populations, such as sex workers, people who inject drugs, and men who have sex with men, who are estimated to make up over one-third of PLH in Ukraine [3]. These numbers fall short of the UNAIDS 95-95-95 targets: 95% of people living with HIV are aware of their status, 95% of those aware receive antiretroviral therapy (ART), and 95% of those on treatment achieve viral suppression.

Identifying and linking PLH to ART is essential if these targets are to be achieved. HIV index testing, which works by identifying potentially exposed partners (named partners) of known PLH (index patients) and connecting those partners with testing, pre-exposure prophylaxis, and HIV treatment services, is a long-standing strategy to achieve this goal [4]. The World Health Organization estimates that 128 nations around the world now have index testing programs, and a growing evidence base supports the effectiveness of this strategy in low- and middle-income settings [5–7]. Such services may be especially beneficial among marginalized and hard-to-reach populations, where legal and social barriers may result in lower uptake of testing and treatment [8]. The WHO considers index testing to be part of a “comprehensive package of testing and care offered to persons with HIV” and the Centers for Disease Control (CDC) and President’s Emergency Plan for AIDS Relief (PEPFAR) have promoted scale-up of index testing in multiple countries, including Ukraine [4].

In Ukraine, scale-up of index testing began in 2018 through PEPFAR support, with the Public Health Center of Ukraine (PHC) naming it as a priority intervention in the 2019–2030 National HIV Testing Strategy [3]. Through this approach, index cases are consented to participation by health facility staff at the time of diagnosis or during routine HIV visits. Index cases then undergo partner elicitation interviews to identify potential named partners, including selecting partner notification preferences and screening for intimate partner violence. However, implementation and scale-up of index testing was not standardized, leading to sub-optimal implementation. In response, the International Training and Education Center for Health (I-TECH), a center in the University of Washington’s Department of Global Health, collaborated with CDC Ukraine and PHC Ukraine to provide targeted financial and technical assistance to 35 health facilities across 11 PEPFAR priority regions (oblasts). Specifically, I-TECH activities sought to strengthen index testing by developing and refining standard operation procedures (SOPs), training 108 healthcare workers on those SOPs as well as counseling skills, supporting program monitoring and evaluation, identifying areas for targeted improvement, and monitoring standards adherence to the SOPs. As part of the intervention, one or two staff at each facility were named as index testing focal persons, with responsibility for case management and follow-up. An analysis found an average case finding of 0.14 per client following the intervention, with the vast majority (90%) of named partners with a positive HIV test being linked to care [9]. Further details on index testing in Ukraine and the technical assistance program have been published elsewhere [9].

Index testing is hypothesized to increase HIV case-finding and to connect people with HIV testing and treatment at an earlier stage of HIV disease progression, reaching partners who might otherwise not seek testing and treatment services on their own [4, 10–12]. However, the extent to which index testing as implemented actually increases the identification of people with HIV– versus just changing how one’s reason for testing is classified– is unknown. Likewise, while the idea that index testing would result in identification of people with HIV earlier in their course of infection is intuitive, limited data exist to support that hypothesis. We investigated two research questions: (1) did ART initiators who came into testing and treatment through index testing tend to initiate treatment at an earlier stage of disease progression compared to those who initiated ART through other pathways into HIV testing and treatment, such as routine HIV testing among pregnant women and TB patients, community-based HIV testing programs, and other pathways and programs; (2) did the index testing program result in an increase in the number of patients newly starting ART.

## Methods

### Study design

This was a retrospective study using patient-level routine infectious disease registry data. To answer the first research question, the study compared indicators of HIV disease stage at ART initiation among named partners identified through index testing services versus other ART initiators, using a non-experimental comparative cohort analysis. To answer the second research question, the study used an interrupted time series (ITS) design to estimate the effect of introducing scaled IT services on the number of ART initiators per month.

### Data sources

The Socially Important Diseases Medical Information System (SID MIS) is a routine, patient-level electronic health system used by all public facilities providing HIV care in Ukraine [13]. It captures data on patient demographics, HIV diagnosis and treatment, clinical factors (e.g., WHO HIV stage), and lab results (e.g., CD4 count) and is filled out by infectious disease doctors and other clinic personnel at the point of service when delivering HIV services [14]. Data on index testing service provision was extracted from an electronic index testing registry (IT registry) developed as part of the technical assistance intervention to enable more robust tracking and reporting of index testing services. Clinicians complete the registry using paper-based patient files either at the point of service or retrospectively. Both the SID MIS and IT registry track patients through a patient-level unique identifier generated by the SID MIS (the MIS ID), enabling merging of the two datasets. For these analyses, SID MIS data were available from October 2018 to May 2021 and IT registry data were available from January 2020 to May 2021. Analysis variables are detailed in Table 1.

**Table 1:** Analysis outcomes and exposure

	Indicator	Definition/Notes
Outcome	Number of new ART initiators	Aggregate number of patients who initiated ART at a given facility in a given month (facility-month).
	Confirmed tuberculosis (TB) diagnosis at the time of ART initiation	Confirmed diagnosis of pulmonary or extrapulmonary TB.
	WHO HIV stage at the time of ART initiation	Defined by the WHO clinical stages of HIV, ranging from stage 1 (asymptomatic) to stage 4 (severe symptoms) [14]. Parameterized as a binary indicator– stage 4 or not.
	CD4 count (cells/mm^3^)	Restricted to lab results obtained within 365 days before or 90 days after date of ART initiation. Parameterized as a binary indicator– CD4 count under 200 cells/mm^3^ or not.
	ART initiation timeliness	Patients without a recorded HIV test date were excluded from analyses involving ART initiation timeliness. Parameterized as a binary indicator– ART initiation within seven days of a confirmatory positive HIV test or not, based on Ministry of Health (MOH) HIV testing guidance.
Exposure	Referral to treatment through index testing services (vs. referral through any means other than index testing; i.e., named partner vs. other ART initiators)	Patients who were referred to treatment through index testing services as a named partner by an index case prior to initiating ART were categorized as named partners. All other ART initiators were categorized as other ART initiators (including those named by index cases but after they had already initiated ART).

### Statistical analysis

Generalized linear mixed models (GLMM) were used for all analyses, the assumptions and limitations of which have been discussed elsewhere [15]. We used marginal parameters from multivariable Poisson regression models with robust error variance to estimate adjusted risk ratios (aRRs) and test the hypothesis that index testing services would identify PLH earlier in their course of infection. Modified Poisson models can provide unbiased estimates for non-rare binary outcomes, where the risk ratio would be overestimated through odds ratios generated through logistic regression and potentially leading to improper interpretation of the results [16, 17]. These models used WHO HIV stage (stage 4 vs. all other stages), CD4 count (below 200 cells/mm^3^ vs. above), and TB status (confirmed TB diagnosis vs. not) at the time of ART initiation as binary dependent variables, with patient identification through index testing services as the primary exposure (i.e., named partners vs. other ART initiators) and controlling for age, sex, and facility type. Similar models were used to assess differences in ART initiation timeliness, using initiation of ART within seven days of a confirmatory positive HIV test as a binary outcome, patient referral to treatment through index testing services (i.e., named partner vs. other ART initiators) as the primary exposure variable, and controlling for age, sex, and facility type, TB status, and WHO HIV stage. To assess changes in ART initiation before and after program implementation, we conducted an interrupted times series (ITS) analysis looking at the number of new HIV diagnoses per month using a negative binomial regression with an AR1 correlation structure to address observed overdispersion and serial correlation, respectively, in the outcome. Data were censored data between October and December 2019 to account for the rolling program rollout period. All models used complete case analysis, included random intercepts for facility to account for clustering, and used a statistical significance alpha of 0.05 for hypothesis testing.

Data were analyzed using R (v 4.1.1) [18]. Regression models were run using the GLMMadaptive (Poisson models) and glmmTMB (ITS models) packages [19, 20].

### Inclusion criteria

Data from patients were included in the ITS analysis if they were: 18 years or older, initiated ART at an intervention facility between May 2018 and May 2021, and were captured in the SID MIS (regardless of engagement with index testing services). The ITS analysis included patients who initiated between October 2018 and May 2021, while the analyses exploring clinical factors and ART initiation timeliness by IT service engagement (i.e., named partners vs. other ART initiators) included patients who initiated ART between January 2020 and May 2021 (i.e., dates for which both SID MIS and IT registry data were available).

### Ethics statement

This study was granted a non-research designation due to minimal risk after review of the evaluation protocol by the University of Washington Human Subjects Division and the US CDC. The Ukraine Ministry of Health Center for Public Health reviewed the protocol and scope of work and provided a letter of support for the project. All data were fully de-identified prior to extraction and use.

## Results

In total, data were drawn from 35 facilities across 11 oblasts (of 24 total in Ukraine) and comprised 14,554 ART initiator patient records from October 2018 to May 2021, of which 5,851 were from the post-intervention time period (January 2020 to May 2021). The following summary statistics are specific to participants who initiated in this post-intervention time period. Over half of included patients were male (58.4%) and aged 25 to 44 (67.9%). The majority of patients presented with a WHO HIV clinical stage of 3 or lower (82.0%) and did not have a confirmed TB diagnosis (89.3%). ART initiation timeliness varied, with most patients (60.1%) initiating ART within seven days of a confirmatory positive HIV test.

A minority of ART initiators in the post-intervention period were named partners (1,024, 17.5%). Almost all named partners were named by a single index case (97.5%), and nearly half also served as index cases themselves (41.8%). Most named partners were linked to index cases through sexual connection (93.0%) rather than needle sharing (7.0%). Named partners were slightly less likely to be male compared to other ART initiators (53.5% vs. 59.4%). Additionally, named partners were less likely to initiate ART at a city or regional AIDS center as opposed to other ART sites (i.e., ART sites based in hospitals, STI clinics, or other primary health care centers) (50.3% vs. 63.5%). Participant characteristics are further detailed in Table 2.

**Table 2:** Participant demographic and clinical characteristics (among patients who initiated ART between January 2020 and May 2021)

	Overall (*n* = 5,851)		Named partners (*n* = 1,024)		Other ART initiators (*n* = 4,827)	
	N	(%)^†^	N	(%)^†^	N	(%)^†^
**Sex**						
Female	2,435	(41.6%)	476	(46.5%)	1,959	(40.6%)
Male	3,416	(58.4%)	548	(53.5%)	2,868	(59.4%)
**Age (mean, SD)**	40.8	(9.2)	41.2	(9.1)	40.7	(9.2)
**Facility type**						
City AIDS Center	672	(11.5%)	94	(9.2%)	578	(12.0%)
Regional AIDS Center	2,910	(49.7%)	421	(41.1%)	2,489	(51.6%)
Other ART site	2,269	(38.8%)	509	(49.7%)	1,760	(36.5%)
**TB status (at time of ART initiation)**						
Confirmed	322	(5.7%)	40	(4.0%)	282	(6.1%)
Suspected	97	(1.7%)	13	(1.3%)	84	(1.8%)
No	5,227	(92.6%)	956	(94.7%)	4,271	(92.1%)
(Missing)	205		15		190	
**WHO HIV stage (at time of ART initiation)**						
Acute HIV infection	13	(0.2%)	3	(0.3%)	10	(0.2%)
Clinical stage 1	2,575	(44.1%)	503	(49.3%)	2,072	(43.0%)
Clinical stage 2	789	(13.5%)	135	(13.2%)	654	(13.6%)
Clinical stage 3	1,408	(24.1%)	233	(22.8%)	1,175	(24.4%)
Clinical stage 4	1,053	(18.0%)	147	(14.4%)	906	(18.8%)
(Missing)	13		3		10	
**CD4 value (at time of ART initiation)**						
>=500	1,293	(25.2%)	229	(25.0%)	1,064	(25.3%)
350–499	984	(19.2%)	198	(21.6%)	786	(18.7%)
200–349	1,170	(22.8%)	203	(22.1%)	967	(23.0%)
<200	1,677	(32.7%)	287	(31.3%)	1,390	(33.0%)
(Missing)	727		107		620	
**ART initiation timeliness**						
Same day	2,033	(36.9%)	466	(47.9%)	1,567	(34.6%)
1–7 days	1,279	(23.2%)	237	(24.4%)	1,042	(23.0%)
8–30 days	825	(15.0%)	164	(16.9%)	661	(14.6%)
31–60 days	157	(2.9%)	23	(2.4%)	134	(3.0%)
61–120 days	68	(1.2%)	10	(1.0%)	58	(1.3%)
> 120 days	1,146	(20.8%)	73	(7.5%)	1,073	(23.7%)
(Missing)	343		51		292	

^†^Percentages were calculated by column (i.e., within each index testing engagement group), and missing observations were excluded from percentage calculations

### Clinical factors

Our multivariable regression models (Table 3) revealed that named partners were less likely to have markers of advanced HIV disease at ART initiation compared to other ART initiators, controlling for age, sex, and facility type. Compared to other ART initiators, named partners were less likely to have a confirmed TB diagnosis (adjusted risk ratio [aRR] = 0.56; 95% confidence interval (CI) = 0.40, 0.78; *p* < 0.001), be categorized as WHO HIV stage 4 (aRR = 0.68; 95% CI = 0.54, 0.85; *p* < 0.001), or have a CD4 count less than 200 cells/mm^3^ (aRR = 0.84; 95% CI = 0.73, 0.97; *p* = 0.015) at the time of ART initiation. Bivariable model results can be found in Supplemental Table 1.

**Table 3:** Multivariable regression of clinical factors at time of ART initiation

	Confirmed TB diagnosis			WHO HIV Stage 4			CD4 count (< 200)		
	(*n* = 5,646)			(*n* = 5,838)			(*n* = 5,124)		
	aRR	95% CI	p-value	aRR	95% CI	p-value	aRR	95% CI	p-value
**Index testing participation**									
Other ART initiators	Ref	-	-	Ref	-	-	Ref	-	-
Named partners	0.56	(0.40, 0.78)	< 0.001	0.68	(0.54, 0.85)	< 0.001	0.84	(0.73, 0.97)	0.015
**Age (years)**	1.02	(1.01, 1.03)	< 0.001	1.03	(1.02, 1.04)	< 0.001	1.03	(1.02, 1.03)	< 0.001
**Sex**									
Female	Ref	-	-	-	-	-	-	-	-
Male	1.48	(1.19, 1.86)	< 0.001	0.92	(0.76, 1.12)	0.416	1.00	(0.92, 1.09)	0.966
**Facility type**									
Other ART site	Ref	-	-	Ref	-	-	Ref	-	-
City AIDS Center	0.40	(0.14, 1.14)	0.087	0.95	(0.56, 1.62)	0.856	0.93	(0.80, 1.08)	0.329
Regional AIDS center	0.55	(0.32, 0.96)	0.035	0.72	(0.47, 1.13)	0.152	0.73	(0.59, 0.90)	0.003

### Timing of ART initiation

As shown in Table 4, as compared to other ART initiators, named partners were more likely to initiate ART within seven days of a confirmatory positive HIV test (aRR = 1.36; 95% CI = 1.23, 1.50; *p* < 0.001), controlling for age, sex, facility type, TB diagnosis, and WHO HIV stage. Bivariable model results can be found in Supplemental Table 2.

**Table 4:** Multivariable regression of ART initiation timeliness

	Timely ART initiation (< 7 days) (*n* = 5,224)		
	aRR	95% CI	p-value
**Index testing participation**			
Other ART initiators	Ref	-	-
Named partners	1.36	(1.23, 1.50)	< 0.001
**Age (years)**	1.00	(1.00, 1.00)	0.659
**Sex**			
Female	Ref	-	-
Male	1.07	(0.99, 1.16)	0.070
**Facility type**			
Other ART site	Ref	-	-
City AIDS Center	1.19	(0.97, 1.46)	0.104
Regional AIDS center	1.27	(1.01, 1.60)	0.042
**TB diagnosis**	0.78	(0.61, 1.00)	0.051
**WHO HIV Stage 4**	0.73	(0.62, 0.86)	< 0.001

### ITS results

**Fig. 1:**
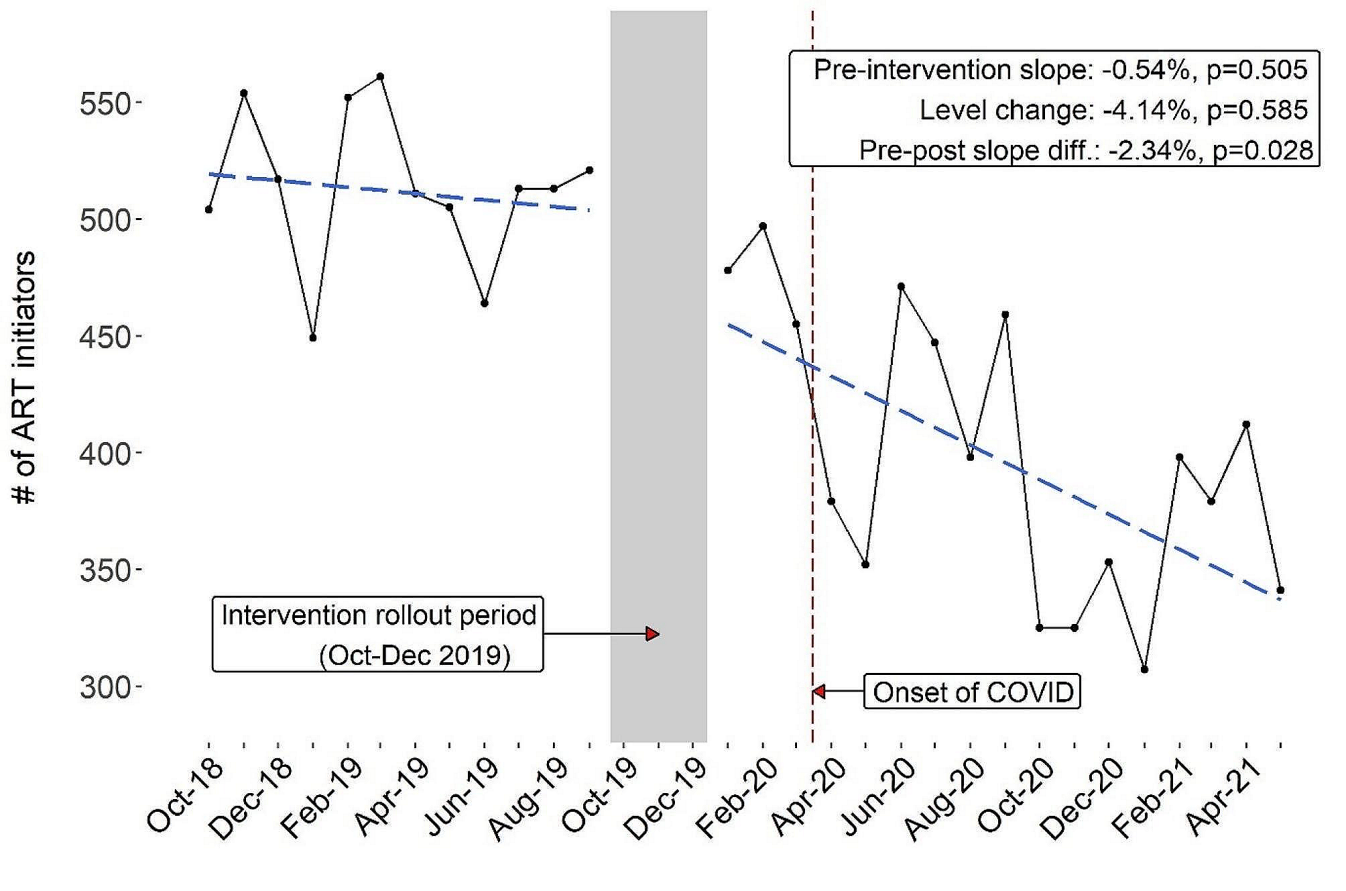
Trends in ART initiation and ITS model results at intervention facilities (October 2018– May 2021). Black dots and line indicate observed ART initiation data. Blue dotted lines represent linear trend from ITS model fitted values

In 2018, there was an average of 525 ART initiators per month, compared to 517 in 2019, 411 in 2020, and 367 in 2021. As shown in Fig. 1, during the pre-intervention period (October 2018 to September 2019), there was a non-significant month-on-month decrease in the number of ART initiators (-0.54%, *p* = 0.505). There was no significant step change in ART initiations comparing directly before to after implementation (-4.14%, *p* = 0.585). The post-intervention time period (January 2020 to May 2021) was associated with a modest additional month-on-month decrease (-2.34%, 95% CI = -0.26%, -4.38%; *p* = 0.028) in mean ART initiations above and beyond what would have been expected based on the pre-intervention trend.

## Discussion

Our analysis found differences in clinical indicators at the time of ART initiation between ART initiators referred to treatment through index testing services (i.e., named partners) and those referred to treatment through any means other than index testing. Named partners were significantly less likely to have a confirmed TB diagnosis, present with stage 4 HIV, or have a CD4 count below 200 cells/mm^3^ at the time of ART initiation. Our results further showed that named partners were significantly more likely than other ART initiators to initiate ART within seven days of a confirmatory positive HIV test. Our findings suggest that index testing services may be beneficial in bringing PLH into treatment at an earlier stage of HIV disease and decreasing delays between HIV testing and ART initiation.

Baseline CD4 count has been shown to be correlated with CD4 recovery, adverse events, and mortality [21–25]. A systematic review and meta-analysis covering 13 observational studies found significantly reduced mortality and progression to AIDS and significantly increased immunologic recovery among ART initiators with baseline CD4 counts greater than 500 cells/mm^3^ [24]. A large-scale, multi-country randomized control trial of 4,685 participants showed that risk of serious AIDS-related events was nearly half among ART initiations with a CD4 county greater than 500 as compared to those with a baseline less than 350 [25]. Studies have also found higher baseline CD4 to be associated with greater life expectancy and higher CD4 counts after initiation [22, 23]. In the context of treatment as prevention, initiating patients on ART earlier in HIV infection (i.e., closer to HIV testing, at earlier HIV stages, and when CD4 values are higher) can also dramatically reduce HIV transmission [26, 27]. This is particularly salient in Ukraine, where prior research has indicated that over one-third of Ukrainian PLH who enrolled in HIV care did so at a late stage of infection [28].

Our findings about the timing of ART initiation in index testing programs are in line with prior research, which has shown similar associations between timely linkage to ART care for patients diagnosed through index testing programs as compared to other testing modalities, perhaps due to the personalized and proactive approach of index testing as well as improved quality of care through additional training providers may receive as part of the index testing implementation [11, 12, 29, 30]. Timely ART initiation can reduce HIV-associated morbidity and mortality and decrease the likelihood of HIV transmission by more quickly achieving viral suppression [31–34]. A Cochrane systematic review and meta-analysis of randomized control trials in LMIC settings found that rapid ART initiation (≤ 7 days from testing) among adult PLH was significantly associated with increased likelihood of both viral suppression and retention in care at 12 months post-initiation [34]. Studies of HIV positive adults in Thailand and West Africa also found significant associations between timely initiation and viral suppression, although neither found early ART initiation to be significantly associated with retention [32, 33].

Although our results are unable to explore likelihood of ART initiation after testing as our sample was limited to just those who initiated ART, our prior research found high levels of rapid linkage to ART (> 90%) among partners newly diagnosed with HIV through index testing services [9]. This suggests that index testing in Ukraine facilitated both earlier testing and diagnosis for partners living with HIV, as well as improved linkage to ART. With an estimated one-in-eight Ukrainian PLH aware of their status but not on treatment, increasing engagement throughout the HIV testing and treatment cascade is vital [1, 2]. This is particularly true given the ongoing war of aggression against Ukraine, which has increased barriers to HIV care, including disruptions to ART provision [35]. While ART is provided without cost through public health facilities in Ukraine, other barriers to the HIV testing and care cascade include lack of HIV knowledge, HIV stigma in the community, lack of psychological support from health workers and transportation barriers [36]. Our research findings suggest that index testing mitigates some of these barriers.

Regarding the ITS results, I-TECH’s collaboration with the MOH to standardize and intensify index testing service implementation occurred at a dynamic time for the health system due to the onset of the COVID-19 pandemic, which greatly disrupted health services shortly after implementation of the program. The WHO reported substantial reductions in both HIV testing (24% reduction in median number of people testing) and ART initiation (15% reduction) in high HIV burden areas in Ukraine in early 2020 after the onset of the COVID epidemic [37]. Uncontrolled ITS analyses assume that the pre-intervention secular trend would have continued if the intervention was not introduced, an assumption that is violated in the presence of history, or events that impact the outcome and happen contemporaneously with the intervention or post-intervention observation period [38]. As these documented disruptions in HIV services aligned with the post- but not pre-intervention period, we suspect that the observed reductions in ART initiations in the ITS model were likely due to the COVID-19 pandemic rather than the intervention itself.

## Limitations

Use of complete case analysis and exclusion of patients who experienced a registration event after initiating ART (e.g., due to a facility transfer) may result in a final analysis sample that is not fully representative of all new ART initiators. However, generalized linear mixed models can provide robust estimates under complete case analysis provided the missingness is ignorable (i.e., missing completely at random or missing at random), although it is not possible to verify if those assumptions hold [39]. A limited number of variables were included in the extracted data; as a result, there may be residual confounding due to unobserved covariates, such as self-stigma related to HIV diagnosis, having friends or family members already on treatment, or education level, which might help explain some of the variation in outcomes. Additionally, we noted differences in ART site type among named partners vs. other initiators, as well as differences in likelihood of markers of advanced HIV disease among ART initiators by site type. While we included ART site type as an adjustment variable in our analytic models, we lacked other data on site attributes and client site preferences to be able to more fully explore the relationship between these variables. This is an area for further research.

## Conclusion

Our analysis showed that named partners, as compared to ART initiators not referred to treatment through index testing services, were significantly less likely to initiate ART with baseline TB diagnosis, HIV clinical stage 4, or CD4 count < 200 cells/mm^3^ and were more likely to initiate ART within seven days of a confirmatory positive HIV test. As baseline opportunistic infections, lower CD4 count, stage 4 HIV, and delayed ART initiation are associated with poorer retention, CD4 recovery, and morbidity and mortality, our findings suggest that index testing services may be beneficial in bringing PLH into treatment at an earlier stage of HIV disease, decreasing delays between HIV testing and ART initiation, and, ultimately, improving patient outcomes.

### Electronic supplementary material

Below is the link to the electronic supplementary material.

Supplemental Tables (bivariate regression results)

## Data Availability

The data that support the findings of this study are available from the Public Health Center of the Ministry of Health of Ukraine (PHC), but restrictions apply to the availability of these data, which were used under agreement with PHC for the current study and so are not publicly available. Data are available from AMS upon reasonable request and with permission of PHC.

## Abbreviations

ART: Antiretroviral therapy
CDC: Centers for Disease Control
I-TECH: International Training and Education Center for Health
IT: Index testing
ITS: Interrupted time series
PEPFAR: President’s Emergency Plan for AIDS Relief
PHC: Public Health Center of Ukraine
PLH: People living with HIV
SID MIS: Socially Important Diseases Medical Information System
