# Supplementary material for: Does HIV index testing bring patients into treatment at earlier stages of HIV disease? Results from a retrospective study in Ukraine
Source: BMC Infect Dis. 2024 Mar 18;24:328. doi: 10.1186/s12879-024-09190-7 (PMC10949801; doi:10.1186/s12879-024-09190-7)
Supplement: Supplementary file 1 — Supplemental Tables (bivariate regression results) [file 12879_2024_9190_MOESM1_ESM.docx]

**Supplemental Materials**

Supplemental Table 1. Bivariable regression of clinical factors at time of ART initiation

|  | **Confirmed TB diagnosis** | | | **WHO HIV Stage 4** | | | **CD4 count (<200)** | | |
| --- | --- | --- | --- | --- | --- | --- | --- | --- | --- |
|  | (n=5,646) | | | (n=5,838) | | | (n=5,124) | | |
|  | RR | 95% CI | p-value | RR | 95% CI | p-value | RR | 95% CI | p-value |
| **IT/PS participation** |  |  |  |  |  |  |  |  |  |
| Other ART initiators | Ref | - | - | Ref | - | - | Ref | - | - |
| Named partners | 0.56 | (0.40, 0.77) | 0.001 | 0.67 | (0.56, 0.81) | <0.001 | 0.83 | (0.73, 0.95) | 0.006 |
| **Age (years)** | 1.02 | (1.01, 1.03) | <0.001 | 1.03 | (1.03, 1.04) | <0.001 | 1.03 | (1.02, 1.03) | <0.001 |
| **Sex** |  |  |  |  |  |  |  |  |  |
| Female | Ref | - | - | Ref | - | - | Ref | - | - |
| Male | 1.50 | (1.20, 1.88) | <0.001 | 0.93 | (0.77, 1.12) | 0.447 | 1.01 | (0.92, 1.10) | 0.857 |
| **Facility type** |  |  |  |  |  |  |  |  |  |
| Other ART site | Ref | - | - | Ref | - | - | Ref | - | - |
| City AIDS Center | 0.41 | (0.14, 1.21) | 0.107 | 0.97 | (0.56, 1.68) | 0.927 | 0.93 | (0.80, 1.07) | 0.294 |
| Regional AIDS center | 0.55 | (0.31, 0.97) | 0.039 | 0.70 | (0.45, 1.09) | 0.115 | 0.69 | (0.56, 0.86) | <0.001 |

Supplemental Table 2. Bivariable regression of ART initiation timeliness

|  | **Timely ART initiation (<7 days)**  (n=5,224) | | |
| --- | --- | --- | --- |
|  | RR | 95% CI | p-value |
| **IT/PS participation** |  |  |  |
| Other ART initiators | Ref | - | - |
| Named partners | 1.38 | (1.24, 1.54) | <0.001 |
| **Age (years)** | 1.00 | (1.00, 1.00) | 0.278 |
| **Sex** |  |  |  |
| Female | Ref | - | - |
| Male | 1.06 | (0.99, 1.15) | 0.106 |
| **Facility type** |  |  |  |
| Other ART site | Ref | - | - |
| City AIDS Center | 1.20 | (0.94, 1.54) | 0.148 |
| Regional AIDS center | 1.28 | (1.03, 1.60) | 0.029 |
| **TB diagnosis** | 0.69 | (0.54, 0.88) | 0.003 |
| **WHO HIV Stage 4** | 0.69 | (0.59, 0.81) | <0.001 |
